# Life Expectancy of White and Non-White Elite Heavyweight Boxers

**DOI:** 10.1007/s40615-019-00656-y

**Published:** 2019-12-03

**Authors:** Thang S. Han, Jonathan Gabe, Pankaj Sharma, Michael E. J. Lean

**Affiliations:** 1 Institute of Cardiovascular Research, Royal Holloway, University of London, Egham, TW10 0EX UK; 2 Department of Endocrinology, Ashford & St Peter’s NHS Foundation Trust, Surrey, UK; 3 Department of Criminology and Sociology, School of Law, Royal Holloway, University of London, Egham, UK; 4 Department of Clinical Neuroscience, Imperial College Healthcare NHS Trust, London, UK; 5 Human Nutrition, School of Medicine, University of Glasgow, Glasgow, UK

**Keywords:** Premature death, Social mobility, Ethnic minorities, Employment, Chronic traumatic encephalopathy

## Abstract

**Background:**

In post-industrial countries, ethnic minorities suffer poorer health and premature deaths. The present study examined ethnic differences in life expectancy and related features among elite heavyweight boxers.

**Methods:**

Dates of birth and death, anthropometry, and championship years were gathered from media archives for champions and challengers (never been a champion) between years 1889 and 2019. Cox regression adjusted for age at contest, nationality, BMI, champion/challenger status, and number of contests was used to assess survival.

**Results:**

All 237 boxers, 83 champions (37.3% whites) and 154 challengers (61.0% whites), who contested for heavyweight championships were identified. By 2019, 110 (75 whites, 34 non-whites) were known to have died. Non-white boxers died at an earlier age than whites boxers (mean ± SD = 59.8 ± 14.2 years versus 67.3 ± 16.4 years, *p* = 0.018) and had shorter survival: HR = 2.13 (95% CI = 1.4–3.3). Among non-white boxers, deaths were higher from neurological disorders: OR = 8.2 (95% CI = 1.3–13.5) and accidents: OR = 15.1 (95% CI = 2.3–98.2), while death from natural causes was lower: OR = 0.2 (95% CI = 0.03–0.8). After boxing careers, fewer non-white boxers had non-manual jobs (34.4% versus 71.8%) than manual (34.4% versus 19.7%) or were unemployed (28.1% versus 2.8%). Reported substance abuse was similar across ethnicity (8.0% versus 8.8%) but conviction rates were higher among non-white boxers (17.6%) than white (1.3%).

**Conclusions:**

Compared with white boxers, non-white boxers tend to die younger with excess neurological and accidental deaths, and they have lower social positions in later life. Sporting authorities should reappraise the wisdom of permitting head injuries in sport and monitor and support the health and wellbeing of sports men and women after retirement.

## Introduction

In most post-industrial countries, members of ethnic minority groups experience a socioeconomic disadvantage [1, 2] which is associated with reduced access to health care, exposure to adverse environments, and consequently poorer health and early death [3–7]. This inequality not only occurs in developed countries but has also been observed in middle-income countries [8, 9]. Education is one route away from poverty while professional sport offers a more rapid alternative [10, 11]. However, professional sports men and women have a short career span and many find transition to a new post-sport occupation challenging [12]. Studies of professional sportsmen at the end of their career have shown that sporting success does not necessarily provide a passage to better occupational opportunities [13]. Indeed, a study of a group of British professional football players has indicated that the majority of these players experienced downward mobility in terms of occupational status and income [14].

Studies have shown that those who are attracted to boxing often come from inner cities and single-parent households, and have limited formal education [15, 16]. Blacks have been disproportionately drawn to boxing for a number of reasons: historically, boxing has been a relatively egalitarian sport where members of different ethnic groups can participate on a fairly equal level; it requires relatively limited facilities and equipment and consequently there tends to be a higher density of boxing clubs in minority neighbourhoods [17]; often, the lack other social opportunities makes boxing attractive to young men from minority groups [18, 19]. Floud et al. [20] argued that professional boxers have tended to come from socially deprived backgrounds where those who grow tallest commonly have better life-opportunities and thus experience upward social mobility. Choosing to become a boxer seems to offer a route to the rapid acquisition of substantial wealth and status [15, 21]. Although there is a body of evidence on racial disparity in mental and physical health, life expectancy [22–24], and mortality [25] among the general population, no previous comprehensive study has assessed how ethnicity could influence the health and life expectancy of these elite boxers once their sporting career is over.

We sought to determine whether there exist ethnic differences in life-course after retirement from boxing, including (1) life expectancy, (2) cause of death, (3) occupation, and (4) substance abuse and convictions in white and non-white male heavyweight boxers who have contested for World Championship titles from 1889 to 2019.

## Methods

### Data Procurement

Data were collected from published media [26], primarily newspapers [27, 28], and supplemented by information from sports magazines, official fight programmes, books, encyclopaedias [29, 30], and publicly monitored websites [31] of all internationally recognised Heavyweight World boxing championships from the first ever official contest (8 August 1889) to the most recent contest (8 February 2019). The present study did not involve patients or the public directly.

### Demographic and Anthropometric Data

Demographic information of male boxers recorded at the time of the championship contests was collected, including date of birth and date of death, cause of death, occupation after boxing career, substance abuse and convictions, champion/challenger status (champions of any of the recognised World boxing titles, principally World Boxing Council, World Boxing Association, World Boxing Organization and International Boxing Federation [32], and unsuccessful challengers who contested but never prevailed in any of the World title bouts), nationality (categorised as North America, Europe, and others), and ethnicity (American white; African-American; European white; and others including mixed race, Hispanic, Samoan, and African) [33]. Weight and height were also collected and BMI calculated as weight/height squared.

### Categorisation of Outcome Measures

Ethnicity was grouped into whites and non-whites; nationality into North America, Europe, and others; and occupation into non-manual, manual, and unemployed. Causes of death include neurological disorders (dementia, stroke), accidents (involving aeroplane, automobile and road traffic, and work accidents), cancers of any aetiology, homicides (murder by any method), metabolic disorders (diabetes, liver and kidney failure), infections (pneumonia, sepsis), cardiac conditions (coronary heart disease, heart failure), and natural causes (due to old age). Reported occupations (day jobs) were classified into non-manual jobs (successful business, managers, public officials, shop ownership, established acting career), manual jobs (doorman, truck driving, training boxers), and unemployed (no day job). Five boxers who died during their boxing career were not included in the analysis in relation to occupational status. Substance abuse includes those who have been reported to have a problem with alcoholism and use of illicit drugs, and convictions include illegal activities that resulted in imprisonment.

### Statistical Analysis

Chi-squared tests were performed to assess differences in the proportion of deaths in different categories of age of death or cause of death between ethnic groups. Cox regression and Kaplan-Meier survival curves were performed to examine the risk of early death and logistic regression causes of death in white (referent group) and non-white boxers. Data were adjusted for age at contest, nationality, champion/challenger status, BMI, and number of boxing matches (or rounds). Analyses were conducted using IBM SPSS Statistics for Windows, version 23.0 (IBM Corp., Armonk, NY, USA). The null hypothesis was rejected when *p* < 0.05.

## Results

### Demographic Features

All 239 boxers who contested heavyweight championships of the World between 1889 and 2019 were identified. Two who were stripped of the titles after being found positive for the use of banned substances were excluded, leaving 237 for analysis. 35.0% were champions and 65.0% were unsuccessful challengers. 65.0% of all boxers came from North America, 18.3% from European countries, and 10.5% from elsewhere. Of these boxers, 52.7% were white and 47.3% non-white. 10.5% of boxers were known to have a history of substance abuse or convictions, 59.6% had non-manual jobs, and 24.0% manual jobs, while 10.6% were unemployed. Neurological disorders (15.5%) and cardiac conditions were among the highest causes of death (16.4%).

The mean (± SD) BMI was 27.8 ± 2.4 kg/m^2^), and mean age at first championship contest was 28.9 ± 4.1 years and at last championship contest was 30.8 ± 4.8 years: mean difference of 1.8 years (95% CI = 1.4–2.3). Among the 211 retired boxers, the median number of boxing matches contested was 53 (IQR = 41–66); there were no ethnic differences (*p* = 0.639): 53 matches by whites and 52.5 matches by non-whites. Similarly, the median number of rounds contested was 318 (IQR = 215–442): 332 rounds by whites and 313 rounds by non-whites (*p* = 0.272). The proportions of those competing for heavyweight titles for the first championship contest at the age of 30–35 years were 24.9%, > 35–40 years were 5.9%, and > 40 years were 1.7%, and the corresponding proportions for the last championship contest were 50.2%, 32.5%, 13.5%, and 3.8%, respectively. The duration of reign as a champion was usually brief, with a median reign of 1.0 year (IQR = 0.6–2.0); only 6% of champion boxers reigned over 4 years. The majority of champions (67.5%) never competed again at the highest level of heavyweight boxing.

### Comparison of Life Expectancy Between Ethnic Groups

By 2019, 110 boxers (75 whites, 34 non-whites) were known to have died (1 white boxer with unknown date of death). Non-white boxers died at an earlier age than whites boxers (mean ± SD = 59.8 ± 14.2 years versus 67.3 ± 16.4 years, *p* = 0.018). In comparison with published data for the general US population, the average life expectancy of white males was 75.7 years and black males was 69.5 years in 2006, and rose to 76.1 years for whites and 71.5 years for blacks a decade later in 2017 [34]. Compared with their respective racial groups in the general US population, non-white boxers had 9.7 years shorter life expectancy and white boxers had 8.4 years shorter life expectancy in 2006, while non-white boxers had 11.7 years shorter life expectancy and white boxers had 8.8 years shorter life expectancy in 2017.

**Fig. 1:**
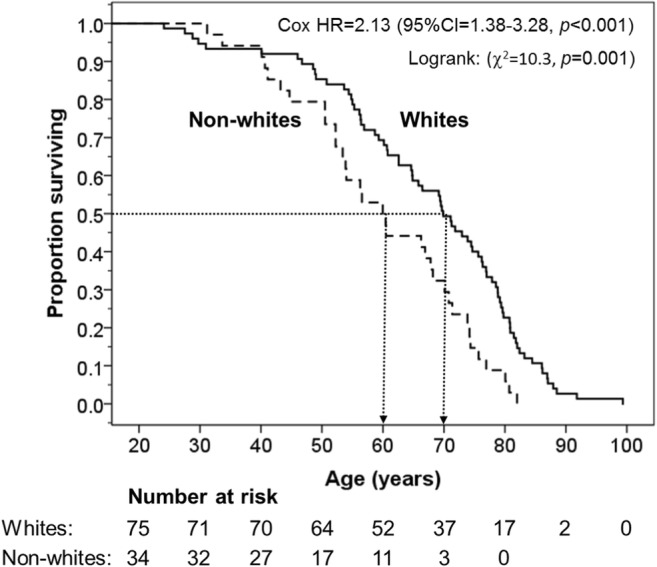
Kaplan-Meier survival plot in 75 white (solid line) and 34 non-white (dashed line) boxers. The dotted lines delineates the median age of the two ethnic groups

Kaplan-Meier survival plot (Fig. 1) shows that compared with white boxers, non-white boxers had a significantly shorter survival: Log rank (Cox-Mantel) test: *χ*^2^ = 10.3, *p* = 0.001, and adjusted hazard ratio = 2.13 (95% CI = 1.38–3.28). Shorter survival was also observed in those with a history of substance abuse: adjusted hazard ratio = 2.28 (95% CI = 1.27–4.10) or with high BMI: adjusted hazard ratio = 2.46 (95% CI = 1.12–5.42) (Table 1).

**Table 1:** Stepwise multivariable Cox proportional hazards regression to assess factors associated with increased hazard of short survival in elite heavyweight boxers. All variables were entered simultaneously as covariates to adjust for each other

	Stepwise Cox regression analysis		
Variables retained by stepwise procedure	Hazard ratio	95% confidence interval	*p*
Ethnicity			
White boxers (reference)	1	**--**	**--**
Non-white boxers	*2.13*	*1.38–3.28*	*0.001*
Substance abuse			
No reported substance abuse (reference)	1	**--**	**--**
Reported substance abuse	*2.28*	*1.27–4.10*	*0.006*
Adiposity			
BMI < 30 kg/m^2^ (reference)	1	**--**	**--**
BMI ≥ 30 kg/m^2^	*2.46*	*1.12–5.42*	*0.025*

Variables eliminated from stepwise procedure: Champion/challenger status, regions, reported convictions, age at contest, boxing career span (or age of retirement of boxing), and number of boxing matches; Italicized values indicate significant association between variables

### Comparison of Causes of Death Between Ethnic Groups

**Fig. 2:**
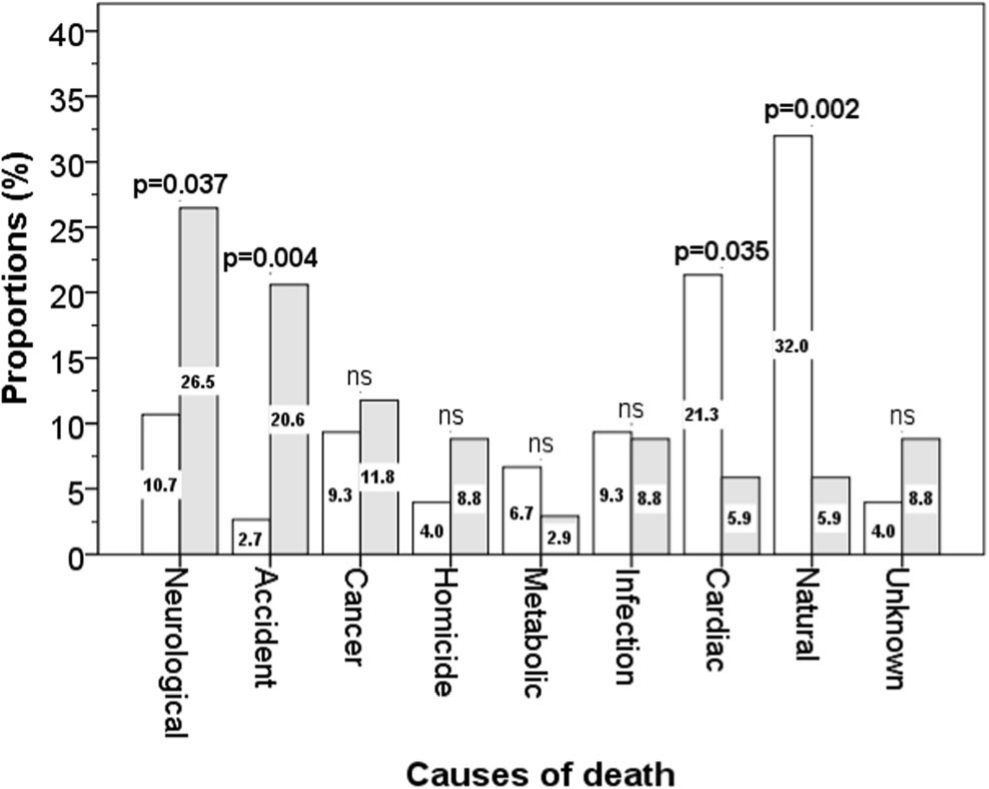
Causes of death in white (open bars) and non-white boxers (grey bars)

Among the 75 white and 34 non-white boxers who had died, the cause of death was mentioned in obituaries for 72 (96%) white and 31 (91.2%) non-white boxers. The proportions of leading causes of death are shown in Fig. 2. There were significant ethnic differences in four of these leading causes of death; compared with white boxers, non-white boxers had significantly higher proportions of deaths from neurological disorders (10.7% versus 26.5%, *p* = 0.037) and accidents (2.7% versus 20.6%, *p* = 0.004), and lower proportions of deaths from cardiac disease (21.3 versus 5.9%, *p* = 0.035) and natural causes (32.0 versus 5.9%, *p* = 0.002). There were no differences for the remaining causes of death (cancer, homicide, metabolic, and infections). Table 2 shows that compared with white boxers, non-white boxers had a higher risk of death from neurological disorders: adjusted odds ratios = 4.18 (95% CI = 1.29–13.51) and death from accidents: adjusted odds ratio = 15.11 (95% CI = 2.33–98.20), while the risk of death from cardiac disorders was lower: adjusted odds ratio = 0.11 (95% CI = 0.01–0.79) and from natural causes: adjusted odds ratio = 0.16 (95% CI = 0.03–0.75).

**Table 2:** Risk of death from different causes in non-white boxers compared with white boxers

	Event rates				Unadjusted			Adjusted for age at contest, substance abuse, conviction, duration of boxing career span, BMI, and number of boxing matches^†^		
	*n*	%	*χ*^2^	*p*	OR	95% CI	*p*	OR	95% CI	*p*
Died from neurological disorders										
White boxers (reference)	8/75	10.7	4.4	0.037	1	**--**	**--**	1	**--**	**--**
Non-white boxers	9/34	26.5			3.06	1.06–8.81	*0.038*	4.18	1.29–13.51	*0.017*
Died from accidents										
White boxers (reference)	2/75	2.7	9.9	0.004	1	**--**	**--**	1	**--**	**--**
Non-white boxers	7/34	20.6			9.95	1.88–49.06	*0.007*	15.11	2.33–98.20	*0.004*
Died from cardiac disorders										
White boxers (reference)	16/75	21.3	4.1	0.035	1	**--**	**--**	1	**--**	**--**
Non-white boxers	2/34	5.9			0.23	0.05–1.08	0.063	0.11	0.01–0.79	*0.029*
Died from natural causes										
White boxers (reference)	24/75	32.0	8.8	0.002	1	**--**	**--**	1	**--**	**--**
Non-white boxers	2/34	5.9			0.13	0.03–0.58	*0.007*	0.16	0.03–0.75	*0.021*

^a^Adjustment for age at retirement from boxing instead of duration of boxing career span did not change the outcomes; Italicized values indicate significant association between variables

### Comparison of Post-Boxing Careers Between Ethnic Groups

**Fig. 3:**
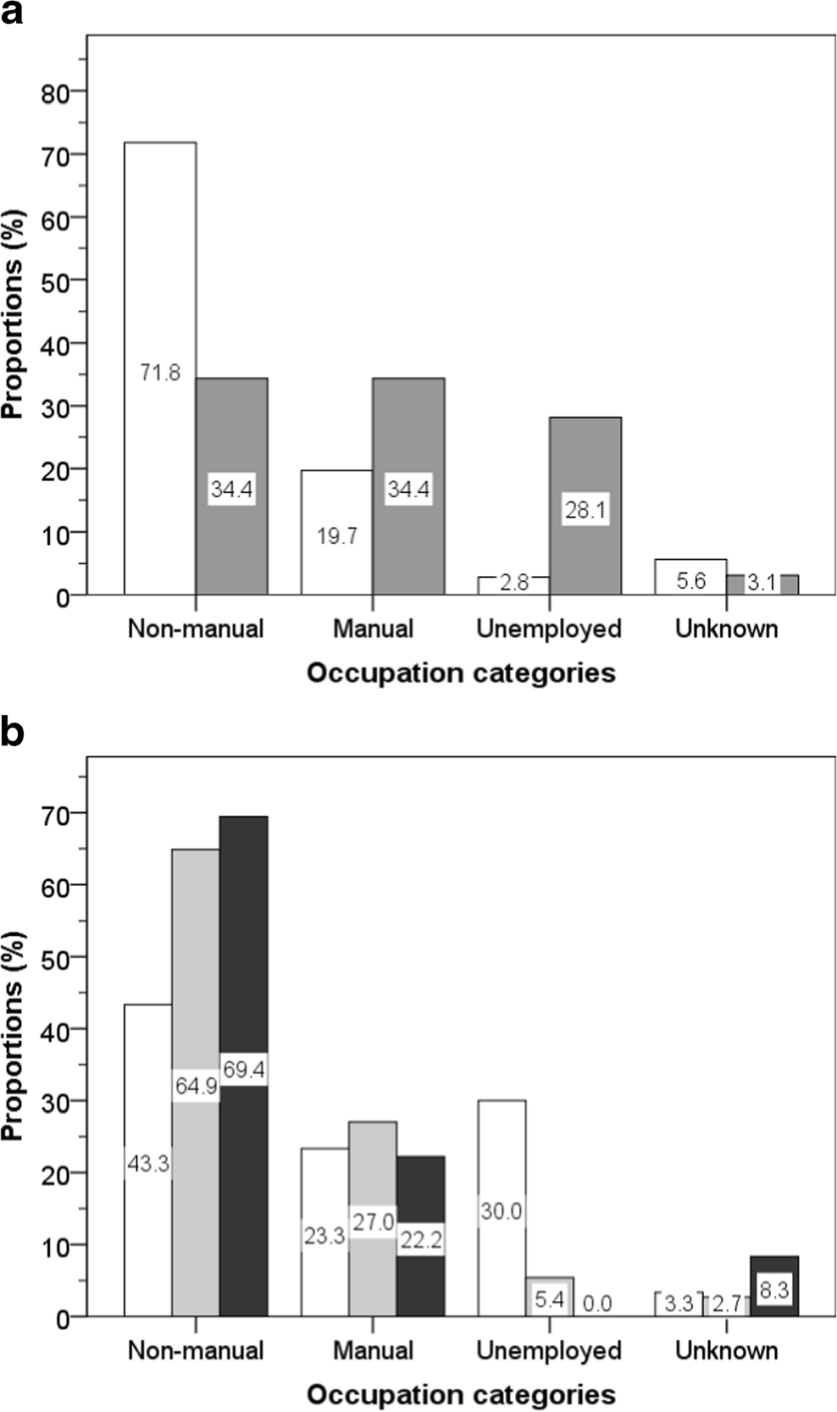
Reported occupations after a boxing career in white boxers (white bars) and non-white boxers (grey bars) (**a**), groups differences: *χ*^2^ = 20.6, *p* < 0.001 and in different tertiles of age of death: before 56.8 years (open bars), 56.8–74.5 years (grey bars), and after 74.5 years (back bars) (**b**), group differences: *χ*^2^ = 18.9, *p* = 0.004

Occupations after the end of boxing career were available for analysis from news archives for 104 deceased boxers (71 white, 33 non-white). Overall, 60.2% of them had had non-manual jobs; 24.3% manual jobs, with 10.7% reported as unemployed; and 4.9% as not known. Among white boxers, 71.8% had non-manual jobs, 19.7% had manual jobs, and 2.8% were unemployed or involved in illegal trades. In comparison, among non-white boxers only, 34.4% had non-manual jobs and 34.4% had manual jobs, while 28.1% were unemployed or involved in illegal trades (Fig. 3a; Table 2).

Among those who died in the lowest tertile of age of death, 43.3% had non-manual jobs, 23.3% had manual jobs, and 30.0% were unemployed. In contrast, among those who lived longest (in the highest tertile of age of death), 69.4% had non-manual jobs, 22.2% had manual jobs, and none were reported as being unemployed (Fig. 3b).

### Comparison of Substance Abuse and Convictions Between Ethnic Groups

**Fig. 4:**
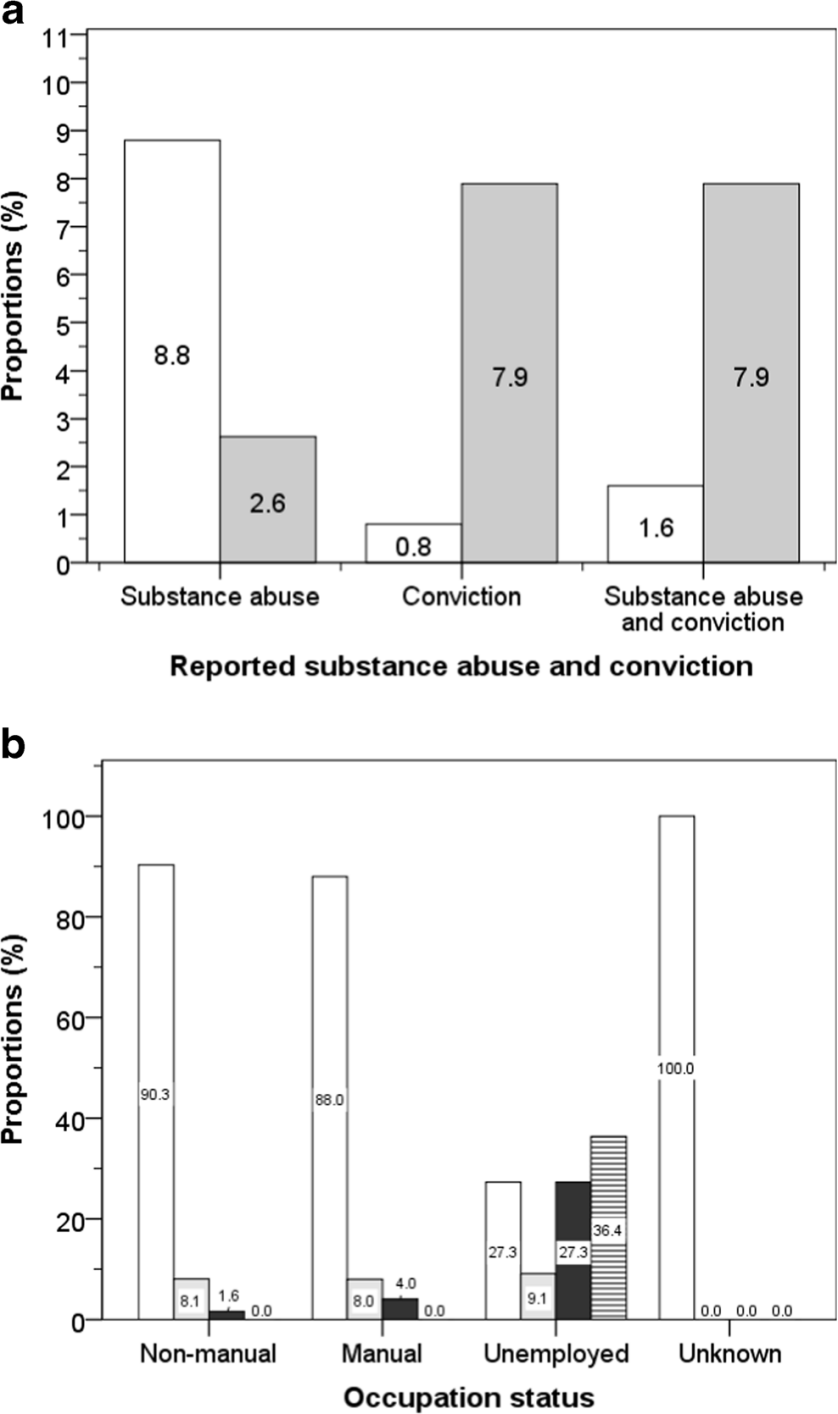
Proportions of boxers reported in the media to have problems with substance abuse or convicted of crimes by ethnic groups (white bars = white boxers and grey bars =n on-white boxers); group differences: *χ*^2^ = 10.5, *p* = 0.005 (**a**), and by occupation (white bars = no reported substance abuse or conviction, grey bars = substance abuse and black bars = conviction of crimes; group difference: *χ*^2^ = 40.1, *p* < 0.001 (**b**)

Based on news archives, a total of 9 boxers (8.3%) were reported to have problems with substance abuse and 7 (6.4%) had a criminal conviction. The proportions of those with substance abuse were similar in white and non-white boxers (8.0% versus 8.8%) but non-white boxers had higher proportions of conviction, 17.6% versus 1.3% (Fig. 4a). Substance abuse was highest among the non-manual group (55.6%) and unemployed boxers (33.3%). Convictions were not reported in the non-manual group, but two convictions were reported in the manual group (8.0%) and five convictions were reported among the unemployed (45.5%) (Fig. 4b).

## Discussion

### Summary

The present study found that among elite heavyweight boxers, non-white boxers died at earlier ages and were more likely to have died from neurological disorders and accidents. Non-white boxers also had higher rates of manual jobs or unemployment and a greater frequency of reported criminal convictions. These findings indicate that despite championship wealth and status, ethnic differences still negatively affected social position and health for non-white boxers once their boxing careers are over. Our findings of ethnic differences in athletes mirror well known disparities in the general populations of the UK [35] and the USA [36].

### Health Consequences of Boxing

Our study has shown that, on average, an elite heavyweight boxer contested 53 matches (318 rounds) during his boxing career (no ethnic differences). Boxing is the only sport that involves intentional repetitive pounding of the head often resulting in concussions and serious brain injuries including chronic traumatic encephalopathy (CTE), also known as dementia pugilistica manifesting as motor, cognitive, and behavioural impairments [37, 38]. CTE has been shown to be associated with atrophy of the thalamus, hippocampi, and caudate [38–40]. Roberts et al. [41] performed a histological examination of the temporal lobe of boxers with CTE and found extensive β-protein plaque deposition in all cases which was comparable to the level observed in Alzheimer’s disease. It is thought that head injury causing axonal, microvascular damage and inflammation may precipitate the neurodegenerative process of CTE [41, 42]. Impaired mental health, a feature of CTE, and involvement with drugs and alcoholism, and the law are also prevalent among boxers. While an excessive number of former boxers suffer from CTE and socioeconomic hardship, assistance from boxing governing bodies does not appear to exist. For example, the British Boxing Board of Control only provides website links for mental health charities such as “Mind” for boxers with depression (www.bbbofc.com/content/rules-boxing-0).

The most striking observations specific to boxers were the increased risk of death from neurological disorders and accidents. These have been shown to exist among ethnic groups in general [43], but the risk appears to be exaggerated for boxers. Studies of patients admitted to hospital with traumatic brain injury has shown that compared with white patients, blacks and Hispanics have a higher mortality [44] and are more likely to be cared for by a junior doctor (resident) on acute admissions [45]. Other studies have found that non-whites were less likely to be insured and therefore less likely to be able to afford rehabilitation [46, 47]. On the other hand, one study has revealed that even having the same insurance coverage (Medicare), blacks and Hispanics were less likely than whites to be transferred to facilities to receive a higher level of rehabilitation [48]. These health care inequalities could therefore explain the higher risk of death from neurological causes among non-white boxers observed in our study.

Cardiovascular disease is more prevalent among white boxers. Its later emergence may reflect their longevity, but their relatively high prevalence may also be related to their underlying body composition and muscle characteristics that enable them to perform this power sport. There is other evidence that people who excel in power sports (as opposed to endurance sports) have increased risk of cardiovascular disease in later life, possibly related to their greater proportion of glycolytic ‘fast-twitch’ type 2B muscle fibres [49].

### Social Inequalities

There may be differences in upbringing between whites and non-whites, which would have influenced on their post-boxing environment. Generally, non-white boxers face greater risk of exposure to adverse environmental factors during their life time, away from boxing. A study on racial victimisation using the National Survey of Americans has revealed that up to a third of blacks had a history of arrest, 18% had been incarcerated, and their frequent exposure to traumas was associated with arrest and imprisonment [50]. We found substance abuse to occur across ethnic groups and occupations while convictions were higher among the non-white group. A number of studies have shown that people who suffer racism are at increased risk of stress-related mental health [22–24] and early death [25]. Williams et al. [23] showed a number of disadvantages among blacks compared with whites including lower education and income, larger household size, lower occupational level, and stress due to racism as well as general stress. These disparities are likely to extend to boxers.

### Post-Boxing Career Opportunities

A boxing career at the highest level is short-lived. We found that on average, the time span during which boxers contested championship titles is 1.8 years, and their reign lasted for just 1 year (median). Half of the boxers’ last contests for world titles were when they were less than 30 years of age; a third were between 30 and 35 years of age; and by the time they passed the age of 40 years, less than 4% were competing for a world title. It is likely that many of the boxers would continue to compete at a lower level (non-title bouts) but most would have retired from boxing before 40eayrs of age and needed to find a new career. World boxing championships are well known to provide the ‘richest prizes in sport’ since the first ever professional contest. In 1910, the purse for the winner was $151,000 (equivalent to $4 million today) [51]. The prize has increased progressively in real terms over the years, with the latest purse for a heavyweight title earned the champion £20 million ($25.9 million) and challenger £6 million ($7.8 million) [52]. As far as we are aware, there have been no report in differences in prize money awarded to white and non-white boxers, but there have been isolated reports including that of a white boxer who fought in the same contest, and lost, but benefited more than the black champion in secondary income such as copyright, e.g. film rights of the fight and sponsorship deals [51]. However, it is known that boxers often have their incomes badly managed [53] and it is possible that this may be more prevalent for non-white boxers. The Muhammad Ali Act in the USA was introduced in 1999 to protect the rights and welfare of boxers [54].

Retiring from boxing is often a sideways move into the training of young boxers (5.8% of our study sample, among whom 50% were non-whites), and it is difficult to obtain reliable estimates of their incomes. A relatively high proportion of boxers (12.6% of our study sample, among whom 69.2% were whites) invested in a sports club as owners or became elected officials in athletics. There was a significant discrepancy in post-boxing employment between white and non-white boxers. About three-quarters of white boxers acquired non-manual jobs, with under 3% being unemployed. In contrast, just over a third of non-white boxers had non-manual jobs while almost 30% were unemployed or involved in illegal trades. Among the underlying reasons for this ethnic disparity, opportunities for higher education and training for new careers after boxing are likely to be important, as are social and environmental factors. We were not able to confirm the existence of support for further education and new career opportunities provided by national and international boxing organisations for boxers undergoing career transition. The lack of assistance for retired athletes is not restricted to boxing. Retired footballers have also demonstrated a rapid decline in physical and mental health [55, 56] with high rates of bankruptcy and divorce, as well as addictions and convictions [57]. Managerial jobs are almost exclusively occupied by whites in the English Premier League; currently, all black managers (7% of all managers in the English Football Leagues) ply their trade in lower leagues. To address this imbalance, the Rooney Rule has been introduced which recommends that at least one black, Asian, and minority ethnic (BAME) candidate should be interviewed for every coaching role. This rule has been adopted by the Football Association but not yet by the Premier League, and only on a voluntary basis by the English Football League [58].

### Post-Boxing Occupations and Health Outcomes

Our findings of the relationship between lower employment and increased risk of death are consistent with previous studies of the general population [59]. Boxing-related health consequences may play a part in the employment profiles and high rates of substance abuse and of criminal convictions among boxers. On the other hand, it is possible that lower employment status (manual occupation or unemployment) contributes to poorer health and earlier deaths in non-white boxers through poverty. Apart from the high risk of injuries related to manual work [60, 61] that may result in disability and death, it is plausible that low level of occupation, which is disproportionally higher in non-whites, leads to early death through poverty [59]; poverty is the fundamental cause of ill health through poor diet and lifestyle habits such as smoking, as well as poorer housing and neighbourhood environment [62].

### Strengths and Limitations

Because of their celebrity status, these elite boxers are uniquely followed and reported on by the media, detailing their future achievements, their unwanted experiences and activities, and their deaths. Most of the boxers included here had published obituaries and biographies, and many have had continuous media coverage that contains a wealth of data, including career and life events as well as date of death. For many, there are publicly monitored resources such as Wikipedia. We also have complete anthropometric measurements for these boxers during their competitive careers, which are analysed in a separate paper [26]. Few other elite sports men and women have such demographic and anthropometric information documented by the media in the same way. There are some potential sources of error and bias, particularly in relation to the use of media reports for scientific analysis. There may be underreporting of bad publicity such as substance abuse and convictions. These may not be disclosed by the boxers themselves, and there is potential for ethnic bias in reporting these by the press if it is largely controlled by whites. Other factors such as smoking and physical inactivity after boxing may also have affected later health but that information was not available.

Despite achieving elite athlete status, non-white heavyweight boxers die at younger ages than white boxers with an excess of neurological and accidental causes of death. Non-whites had poorer social profiles in later life after their boxing careers with more substance abuse and criminal convictions. Sporting authorities should reappraise the wisdom of permitting blows to the head in sport and should monitor and support the health and wellbeing of sports people after retirement.
